# High-grade serous carcinoma of unknown primary origin associated with STIC clinically presented as isolated inguinal lymphadenopathy: a case report

**DOI:** 10.3389/fonc.2023.1307573

**Published:** 2024-02-02

**Authors:** Paola Giancontieri, Camilla Turetta, Giacomo Barchiesi, Angelina Pernazza, Gemma Pignataro, Giuliano D’Onghia, Daniele Santini, Federica Tomao

**Affiliations:** ^1^ Department of Radiological, Oncological and Anatomo-Pathological Science, Policlinico Umberto I, Sapienza University of Rome, Rome, Italy; ^2^ Department of Maternal and Child Health and Urological Sciences, Sapienza University of Rome, Rome, Italy; ^3^ Department of Surgery, Sapienza University of Rome, Rome, Italy

**Keywords:** STIC, HGSOC, ovarian, cancer, lymphadenopathy, metastasis, p53

## Abstract

Serous tubal intraepithelial carcinoma (STIC) is a precancerous lesion of high-grade serous ovarian carcinoma (HGSOC). Usually, it arises from the fimbrial end of the tube, and it is associated with metastatic potential. On average, the time to progress from STIC to HGSOC is 6.5 years. Therefore, whenever a STIC lesion is found, surgical staging and prophylactic salpingectomy are recommended in order to prevent ovarian cancer. We report a rare case of a 45-year-old female patient who clinically presented an isolated right inguinal lymphadenopathy. The remaining clinical examination was normal. Therefore, an excisional biopsy of the lymph node was performed. Pathological analysis revealed a high-grade serous carcinoma, most likely of gynecological origin. Due to histological evidence, a computed tomography (CT) scan was carried out. There was no CT evidence of ovarian disease, pelvic involvement, intra-abdominal lymphadenopathies, metastatic disease, or ascites. All tumor markers were negative. The patient underwent laparoscopic hysterectomy and bilateral salpingo-oophorectomy followed by surgical staging. Surprisingly, pathological examination showed a STIC lesion in the fimbria of the left fallopian tube. We aim to report the potential capability of STIC to spread particularly through lymphatic pathways rather than peritoneal dissemination.

## Introduction

Ovarian carcinoma is the leading cause of death among gynecological malignancies (1). Due to the usual lack of symptoms associated with early-stage disease, most of the cases are diagnosed when the cancer has already progressed. This is one of the major contributing factors to the high mortality of this disease. The prognosis and treatment response depend on the stage, grading, and histological subtype of the tumor (1).

Ovarian cancer is known to be associated with BRCA 1 or 2 mutations. In these cases, the tumor tends to respond better to chemotherapy than a BRCA wild-type (WT) tumor at the same stage and grading, therefore showing a more favorable survival outcome (2).

High-grade serous ovarian cancer and the endometrioid subtype are sensitive to platinum-based chemotherapy, whereas low-grade serous ovarian cancer, mucinous cancer, and clear cell cancer are less sensitive to these regimens. Because of this resistance to systemic therapy, primary surgical resection has been shown to have a larger impact on reducing tumor burden in these subtypes (3, 4).

A large part of high-grade serous ovarian carcinomas (HGSOCs) seem to arise from the distal fimbrial end of the fallopian tube from a precursor lesion known as serous tubal intraepithelial carcinoma (STIC). STIC lesion happens when normal fallopian tube epithelium is substituted by atypical non-ciliated cells with immunohistochemical and morphological aspects of HGSOC with no invasion of the underlying stroma. Despite the absence of stromal invasion, the cells of STIC can exfoliate from the tissue and eventually spread, resulting in a disseminated HGSOC (5).

**Figure 1:**
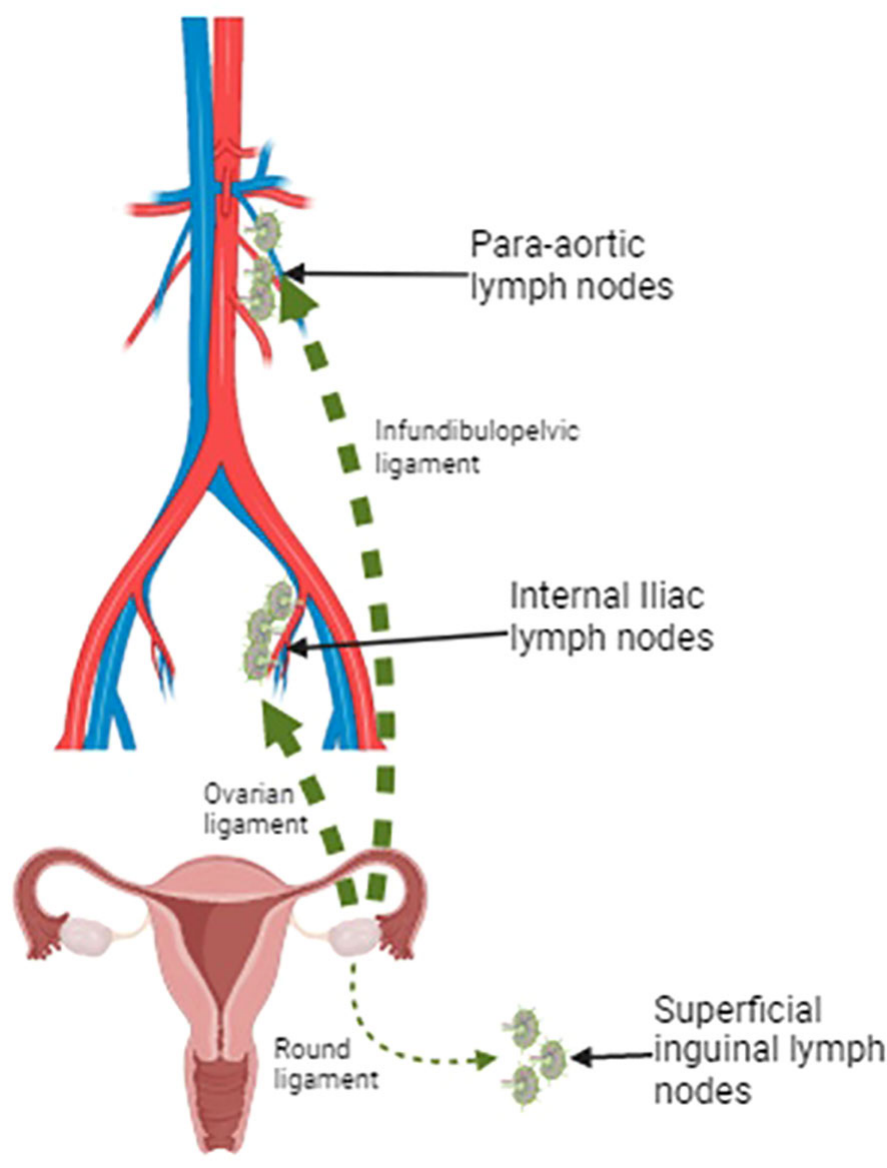
Lymphatic drainage pathways of the ovaries: the two major pathways include the lymphatic drainage via infundibulopelvic ligament toward para-aortic lymph nodes and the lymphatic drainage via the ovarian ligament to the obturator lymph nodes and the internal iliac artery. A minor drainage route goes through the round ligament and reaches the inguinal lymph node. Created with BioRender.com.

Lymphatic spread of ovarian carcinoma usually involves para-aortic and retroperitoneal lymph nodes (**Figure 1**). Isolated secluded inguinal lymph node metastasis is an uncommon manifestation of ovarian cancer. In most patients, a primary tumor was identified by either accurate imaging or a diagnostic surgical procedure (6, 7).

In this paper, we describe a rare case of a woman who presented a right inguinal lymph node metastasis of HGSOC, with unknown primary origin, that was discovered to be associated with a STIC in the fimbrial region of the left fallopian tube.

## Case presentation

A 45-year-old woman was referred for an enlarged right inguinal lymph node. Her medical history consisted of Hashimoto’s thyroiditis, linear scleroderma, and chronic headaches. She had only one birth (primiparous); she had a spontaneous menopause at the age of 39 years, followed by hormone replacement therapy with levonorgestrel and ethinyl estradiol. A family history of oncologic disease was negative. She did not report pain, erythema, or ulceration in the inguinal area in the previous 2 months.

In February 2023, clinical examination confirmed the presence of an enlarged lymph node, measuring approximately 20 × 15 mm, hard consistency, and adherent to underlying tissues, suspicious for heteroplastic disease. Laboratory blood tests were normal with tumor marker levels of CA125 at 18.9 U/mL, Ca15-3 at 24 U/mL, Ca 19-9 at 8.56 U/mL, and carcinoembryonic antigen (CEA) at 1.2 ng/mL. Alpha-fetoprotein (AFP), human epididymis protein 4 (HE4), and beta-human chorionic gonadotropin (beta-HCG) were negative (respectively 2.9 ng/mL, 59.9 pmol/L, and 5.0 mUI/mL).

Therefore, an excisional biopsy of the lymph node was performed. Pathological examination revealed neoplastic cells with marked cytologic atypia, organized in solid sheets or slit-like spaces. On immunohistochemistry, the neoplastic cells were diffusely positive for CK7, PAX8, WT1, P16, and estrogen receptors (ERs) and negative for p40, GATA3, CDX2, CK20, and TTF-1; p53 exhibited an abnormal pattern expression (negative staining). Based on the morphological and immunohistochemical findings, a diagnosis of lymph node metastases from high-grade serous carcinoma most likely of tubo-ovarian origin was made.

Following the resection of the lymph node and the pathological results, a full-body computed tomography (CT) scan was requested. It showed a rounded formation (16 × 15 mm) of regular and sharp margins, with the typical density of the soft tissues, in the left retroperitoneal site between the spleen and left kidney, closely adherent to the posterior diaphragmatic profile. No other abnormalities were found. Therefore, a positron emission tomography–CT scan (PET-CT scan) was requested. No pathological uptakes were detected.

The patient underwent a primary cytoreduction, consisting of hysterosalpingo-oophorectomy, partial omentectomy, and resection of the diaphragmatic nodule.

**Figure 2:**
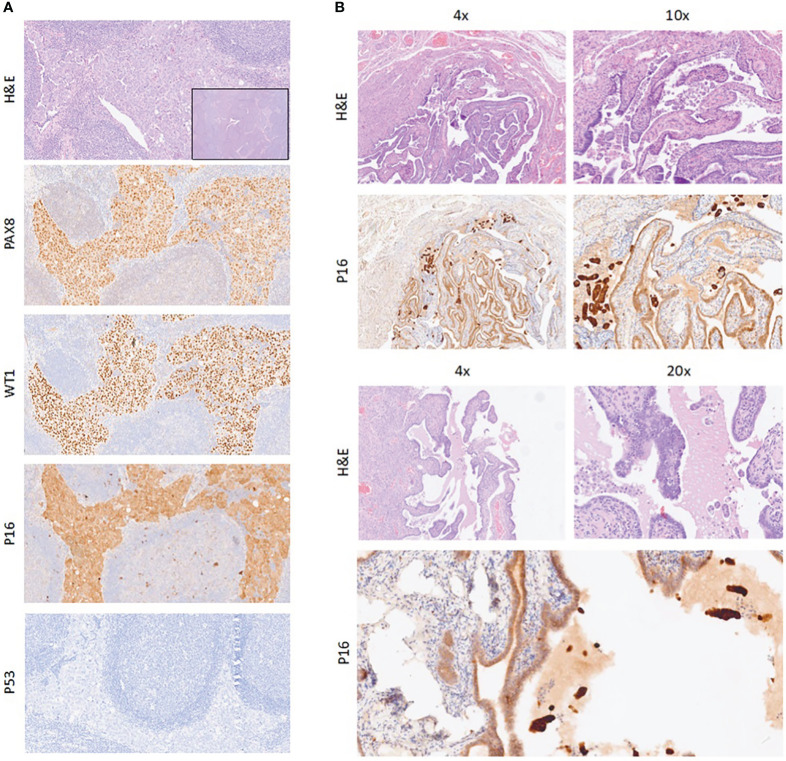
**(A)** Neoplastic cells in lymph node (H&E); on immunohistochemical stains, neoplastic cells are positive for PAX8, WT1, and p16 and negative for p53. Magnification, ×10. Inset: magnification, ×4. **(B)** Sections of fallopian tube with STIC and small groups of neoplastic cells exfoliated in the lumen (H&E); on immunohistochemical staining, neoplastic cells are positive for p16. Magnification, ×4, ×10, and ×20. STIC, serous tubal intraepithelial carcinoma.

Neither microscopic nor macroscopic neoplastic lesions were found at the pathological examination; based on hematoxylin and eosin-stained slides, the pathologist found a microscopic focus of STIC in the fimbria of the left fallopian tube (**Figure 2**). The epithelium of the lesion showed some degree of stratification, and its cells were characterized by irregular luminal borders; small groups of exfoliated neoplastic cells were found in the fallopian tube lumen, near the STIC focus. Multiple sections were made to exclude stromal invasion. The immunohistochemical features were similar to those observed in the lymph node metastases. No other sites of disease were found.

Finally, the case was considered as an occult non-invasive tubal carcinoma (STIC) presenting with a distant inguinal lymph node metastasis. Additional investigation showed no BRCA 1 or 2 mutations. Homologous recombination deficiency (HRD) status assessed by SOPHiA DDM Dx HRD Solution^®^ was undetermined.

**Figure 3:**
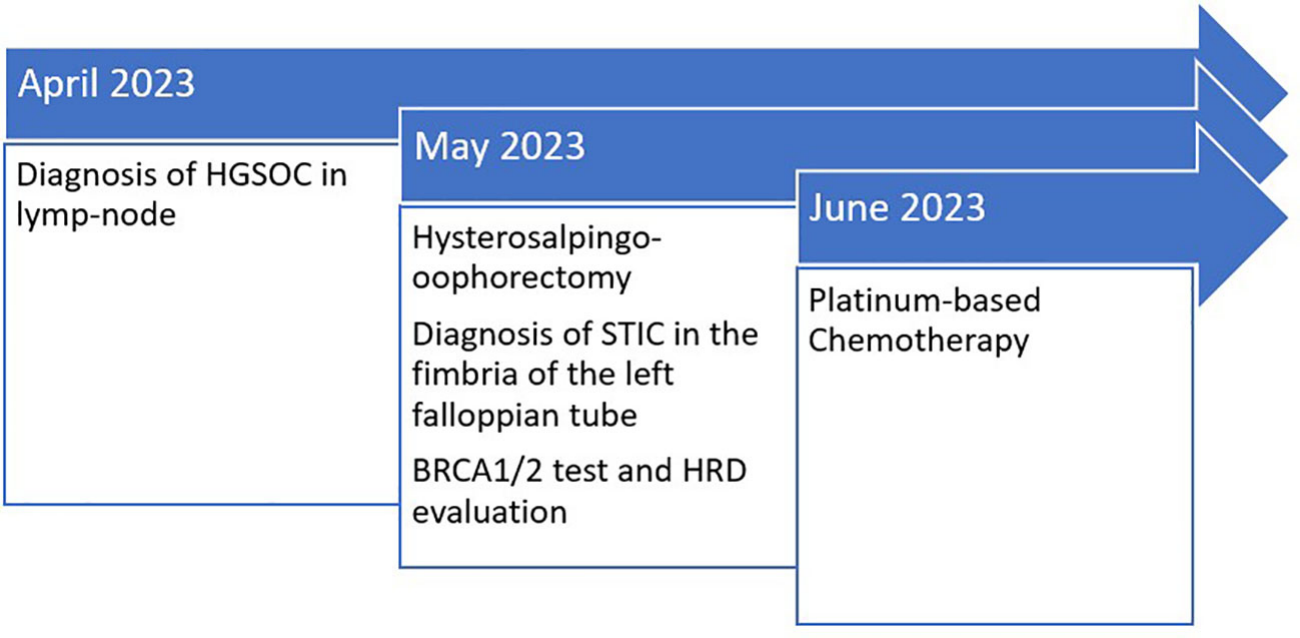
Timeline table.

According to the decision of the gynecological oncology multidisciplinary team, the patient was scheduled for six cycles of weekly carboplatin–paclitaxel-based chemotherapy every 3 weeks (**Figure 3**).

## Discussion

This case shows a HGSOC diagnosis clinically presented with a single inguinal lymphadenopathy in the absence of heteroplastic lesions in the uterus and ovaries. The rarity of this case is the singular presence of a STIC lesion in the fimbria of the left fallopian tube with no other concomitant lesions. Patients with ovarian cancer often present with metastatic disease (8). HGSOC appears to arise from either the ovarian surface epithelium or the fallopian tube epithelium. To establish the site of origin, extensive examination of the adnexa is required (ovaries, fallopian tubes, and their fimbriae) (9).

Isolated inguinal lymph node metastasis is an uncommon manifestation of ovarian carcinoma. Only a few cases in which inguinal lymphadenopathy was the clinical manifestation of an epithelial ovarian tumor were described in the medical literature (7, 10–15).

However, lymphatic involvement is usual in ovarian carcinoma; it is reported in approximately 14%–70% of patients and mostly in the pelvic and para-aortic areas (6) (see **Figure 1**).

According to Kleppe et al. work (10), the possibility of a rare inguinal nodal involvement from ovarian carcinoma is based on two lymphatic drainage pathways. The two major pathways include the lymphatic drainage via the infundibulopelvic ligament toward para-aortic lymph nodes and the lymphatic drainage via the ovarian ligament to the obturator lymph nodes and the internal iliac artery. A minor drainage route goes through the round ligament and reaches the inguinal lymph node; this could explain the inguinal lymph node involvement in the absence of para-aortic or pelvic lymphadenopathies.

It has been postulated that previous abdominal surgery may lead to anatomical modifications that could favor the spread of the tumor to the groin region (11, 12). It could be assumed that in these patients, previous surgery could have a role in the tumor spread. However, in our case, the patient did not undergo intestinal or gynecological surgery before diagnosis.

About the case we report, the only finding at the histological examination after surgery was a STIC focus in the fimbria of the left fallopian tube. We suggest that this may be the precursor of HGSOC metastases in the inguinal lymph node.

Clinical evidence supported the hypothesis that STICs can arise from epithelial cells of the fallopian tube and transform into HGSOC by rapidly disseminating to involve the ovarian and peritoneal areas (16).

STIC are lesions with p53 mutations and increased proliferative capacity, and they are observed in at least 60% of women with HGSOC of the ovary and/or peritoneum (16). p53 aberrant expression may be defined by three different patterns: 1) strong and diffuse staining in at least 80% of cells, 2) no expression (with an intact internal control), and 3) cytoplasmic staining with weak nuclear staining (rare) (17).

Clinical evidence supports the hypothesis that STICs can arise from epithelial cells of the fallopian tube and transform into HGSOC by rapidly disseminating to involve the ovarian and peritoneal areas (16). Given the ability of STIC to spread beyond the fallopian tube without invasion of underlying stroma, the term carcinoma *in situ* should be abandoned, as it implies that there is no potential for metastasis. Histologically, STIC is the earliest morphologically recognizable form of tubal carcinoma. STIC is characterized, as previously stated, by the absence of invasion of underlying fallopian tube stroma and by the presence of cytologic abnormalities, which give the involved epithelium a darker appearance at low-power magnification compared with the adjacent normal epithelium. In cases with invasive carcinoma in the same tube, STIC may be found directly adjacent to the invasion.

A meta-analysis of 3,121 patients with BRCA 1/2 pathogenic variant who underwent risk-reducing salpingo-oophorectomy (RRSO) showed that the 5- and 10-year risk to develop peritoneal carcinomatosis (PC) was 10.5% and 27.5%, respectively, in patients with STIC, whereas the corresponding risk was 0.3% and 0.9%, respectively, for women without STIC at RRSO (18). Among them, 11 women with STIC who received chemotherapy did not develop PC. Four patients received chemotherapy because of positive peritoneal washing; the other seven patients received chemotherapy depending on a subjectively increased risk of PC, age at RRSO, or the histologic features of the STIC itself. Considering that chemotherapy has serious adverse effects, additional prospective evidence should be provided before this treatment is recommended, evaluating its benefits by each case and the data of the results this treatment had on PC risk throughout follow-up (18).

Przybycin et al. study (19) documented STIC in 61% of sporadic advanced HGSOC submitted for histologic examination through a specific protocol for sectioning and extensively examining the fimbrial end of the fallopian tube (SEE-FIM protocol).

The clonal relationship between STIC and concurrent HGSOC may be investigated by genomic analysis. Mutational evaluation of pelvic HGSOC with concomitant STIC has revealed that both lesions had identical TP53 mutations in most cases (20, 21). TP53 gene encodes a tumor suppressor protein containing transcriptional activation, DNA binding, and oligomerization domains. Approximately 97% of extrauterine HGSOCs exhibit TP53 mutation (22).

Since finding the same mutation that occurs simultaneously in different sites is extremely rare, matching the TP53 mutation in different locations is clear evidence of clonal identity (20–22).

According to Singh N. et al., most extrauterine high-grade serous cancers arise in the distal fallopian tubes rather than the ovary, developing from STIC, a small precursor lesion (23).

A retrospective study of 231 patients detected STIC in 68.4% of all HGSOCs. Specifically, only two of them (1.26%) were affected by pelvic and para-aortic nodal metastases without any other intra-abdominal involvement, while in the majority of women, peritoneal spread was present (13.9%) (24).

Only three cases of HGSOC presenting with isolated inguinal lymph nodes with unknown primary origin have been published (**Table 1**).

**Table 1:** Reported cases of inguinal lymph node metastasis of HGSOC with unknown primary site origin.

Author	Age	First diagnosis	Side	Histology	CA125	Previous abdomen surgery
Carrabin et al. (13)	59	Inguinal lymph node mtx	Right	Borderline tumor	/	Appendicectomy
Dam et al. (14)	62	Inguinal lymph node mtx	Right	HGSOC	3,628	Hysterectomy
Restaino et al. (15)	78	Inguinal lymph node mtx	Right	HGSOC	/	Hysterectomy

HGSOC, high-grade serous ovarian carcinoma; mtx, metastasis. The symbol “/” means “not reported”.

Carrabin et al. (13) supported the possibility of the presence of ectopic ovarian tissue because the tumor was completely surrounded by normal ovarian tissue at the final histological examination. Dam et al. (14) described a patient with a history of hysterectomy for benign pathology, with an enlarged inguinal right lymph node; after surgery, a diagnosis of a nodal metastasis of a serous high-grade papillary cancer, most likely with ovarian origin, was made. Bilateral salpingo-oophorectomy was performed, but pathological examination could not identify a primary tumor. Restaino et al. (15) described a case of a 78-year-old woman who was brought to the physicians’ attention because of an enlarged right inguinal lymph node. The diagnosis of metastasis from HGSOC was made. The patient underwent bilateral salpingo-oophorectomy and peritoneal biopsies. The final pathology examination did not reveal any evidence of disease. The patient received six cycles of carboplatin and paclitaxel. After 1 year, the patient developed a left inguinal enlarged bulky node of 4 cm recurrence.

To explain the absence of a primary site cancer, some authors suggest that the immune defense mechanisms of the host destroyed the primary tumor without affecting the lymphatic metastasis (25).

Finally, diffuse and strong p16 expression may be expressed in STIC lesions with similar patterns in many HGSOCs.

In the present case report, both STIC and lymph nodal lesions had the same p53 aberrant pattern (consisting of loss of expression—p53 null mutation) and p16 positive staining.

These pathological findings support the hypothesis of the origin of the metastatic lymph node from the STIC as reported in this paper. Notably, considering the exfoliation process and the non-invasive attitude of STIC, we expected a peritoneal dissemination rather than a lymphatic spread.

## Conclusions

According to the literature, very few cases reported inguinal metastasis of HGSOC with unknown primary origin. Isolated ovarian metastases of inguinal lymph nodes remain rare.

Reviewing the literature on this topic, the hallmark of this case is the presence of concomitant STIC lesion and distal nodal disease in the absence of pathological adnexal or peritoneal neoplastic involvement.

Moreover, immunohistochemistry and morphological features suggested that STIC and HGSOC are not linked to a primary tumor, raising the hypothesis that STIC might be considered not just a simple precursor of ovarian cancer but a carcinoma with a capacity for metastasizing.

## Data availability statement

The raw data supporting the conclusions of this article will be made available by the authors, without undue reservation.

## Ethics statement

Written informed consent was obtained from the individual(s) for the publication of any potentially identifiable images or data included in this article. All procedures involving human participants were performed in accordance with the ethical standards of the institutional and/or national research committee and the 1964 Declaration of Helsinki and its later amendments or comparable ethical standards. This article does not contain any research analysis on humans or animals.

## Author contributions

PG: Conceptualization, Investigation, Writing – original draft. CT: Data curation, Writing – review & editing. GB: Conceptualization, Validation, Writing – review & editing. AP: Data curation, Writing – review & editing. GP: Data curation, Writing – review & editing. GD: Resources, Writing – review & editing. DS: Supervision, Writing – review & editing, Validation. FT: Formal analysis, Methodology, Writing – review & editing.
